# The Sertindole Safety Survey: A retrospective analysis under a named patient use programme in Europe

**DOI:** 10.1186/1471-244X-8-57

**Published:** 2008-07-18

**Authors:** Christophe Lançon, Mondher Toumi, Christophe Sapin, Karina Hansen

**Affiliations:** 1 Département de Psychiatrie, CHU Sainte Marguerite, Marseilles, France; 2 Université de Lyon I, Lyon, France; 3 H. Lundbeck A/S, Paris, France

## Abstract

**Background:**

After sertindole's suspension, health authorities established a specific named-patient use (NPU) programme in order to supply sertindole to patients who did not respond to or did not tolerate alternative treatments. This programme provided the possibility of prospectively following an exhaustive cohort of patients treated with sertindole after its suspension. A survey was performed to assess sertindole's modalities of prescription, assess and document any serious adverse events (SAEs), and assess the mortality rate within the NPU cohort.

**Methods:**

The study comprised a survey of sertindole-treated patients in eleven European countries. All patients treated with sertindole within the NPU programme were eligible for the study.

**Results:**

1,432 patients were included in the study. The reason for sertindole prescription was lack of efficacy (approximately 50%) or adverse events (approximately 20%) of other antipsychotic treatments. The mean sertindole dose was 13.4 mg daily. Lack of efficacy and adverse events were reported as reasons for sertindole discontinuation.

A total of 97 SAEs were recorded, including ten fatal outcomes, which occurred during the study period or within thirty days after sertindole discontinuation. The all-cause mortality rate was 0.51 per 100 Person-Years of Exposure (95% Poisson confidence interval: 0.23–0.97). QTc prolongation was reported in 15 patients (1.05% of total patients), being a rate of 0.85 per 100 Person-Years of Exposure [95% CI: 0.48–1.41].

**Conclusion:**

Although prescribing and supplying sertindole were subject to administrative constraints, a significant number of patients were treated with sertindole, thus supporting the need for sertindole in specific cases.

**Trial registration number:**

Not applicable.

## Background

Schizophrenia is a severe and frequent chronic psychosis, present in all cultures [1,2] with a prevalence of approximately 1% of the population during their lifetime [3,4] and an incidence ranging from 16–42 new cases per 100000 [5].

The onset of the disease mostly occurs during young adulthood (mid 20 s for men, late 20 s for women), although onset can be earlier or later [6]. Age of onset for women is 3–6 years later than in men [7]. The disease course and outcome of schizophrenia are extremely variable among patients, and the presentation of the disorder changes over time within individuals. In many patients, the disorder is cyclical with acute exacerbations and remissions. A small number of patients remain chronically ill throughout their life [8].

Patients with schizophrenia tend to have a reduced quality of life. They are also at greater risk than the general population for substance abuse, concomitant mental disorders, illness from general medical conditions and premature death from suicide, accidents and natural causes. 50% of patients with schizophrenia may attempt suicide at one time in their life, and at least 10% die due to suicide [8].

Despite progress in the management of schizophrenia following the introduction of atypical antipsychotics in the late nineteen nineties, current pharmacological options still carry some limitations. The safety advantages of the atypical antipsychotics have been questioned because of their propensity to induce weight gain [9] and alter glucose and lipid metabolism [10,11]. Metabolic disorders associated with atypical antipsychotics were reiterated in a recent, large, pragmatic clinical trial: The Clinical Antipsychotic Trials of Intervention Effectiveness (CATIE) study conducted in the US [12].

Sertindole is an atypical antipsychotic with a good tolerability profile [13], that may favour long-term treatment adherence [14], reduce rates of re-hospitalisation [15] and suicide [16], and improve overall functioning [14]. It has been associated with expected QT prolongation but has no increased risk of all-cause mortality. It was marketed in the UK in 1996, and marketing was extended to the rest of Europe in 1997. However, an excess relative reporting of cases suggestive of sudden death led to the manufacturer suspending drug supply on November 2^nd ^1998, pending further assessment of the risk [17]. A Named Patient Use (NPU)/compassionate use programme was set up in individual countries to allow well-controlled patients to stay on sertindole treatment during the suspension. The European Committee for Proprietary Medicinal Products (CPMP) issued a temporary market suspension in January 2000 and asked for complementary benefit/risk evaluation of the product. The present survey was part of this evaluation, and aimed to specifically assess the benefits and risks associated with sertindole use. The Sertindole Safety Survey offered a unique opportunity to analyse sertindole prescription patterns, assess serious adverse events and determine mortality rates within an exhaustive cohort comprised of patients receiving sertindole via the NPU.

## Methods

### Study design and objective

The sertindole safety survey (SSS) was a multicenter retrospective survey of all patients treated with sertindole in Europe after its market suspension. The survey was conducted in Austria, Belgium, the Czech Republic, Estonia, Finland, Germany, Hungary, Latvia, The Netherlands, Norway and Switzerland. In Greece, Denmark, Luxembourg, and in the UK only a small number of patients (less than 10) entered the NPU programme. No patients were treated with sertindole after the market suspension in Ireland, Italy, Portugal and Spain. Sertindole was not approved in France and Sweden at the time of its market suspension in Europe. The objectives of the survey were to assess sertindole prescription patterns in a particular population, assess and document serious adverse events, and assess mortality.

### Patient selection

Specific prescription modalities of the NPU programme allowed most, if not all, patients to be identified in each country. All available sources were used to identify sertindole prescribers as exhaustively as possible. In Austria, Belgium, Finland, Hungary and Switzerland this was done using the national health authority's records. In the Czech Republic, Estonia and Norway, all psychiatrists were contacted and invited to participate in the study. In Latvia, all known sertindole prescribers were contacted. In Germany and Hungary, records were used from the "Controlled Use after the Suspension of Marketing of Sertindole" and the "Serdolect Safety Survey Hungary" programmes respectively. Both of these programmes had been approved by the national health authorities. In Belgium, Estonia and Hungary the survey was exhaustive, in the other countries 70 to 95% of patients were identified.

### Inclusion criteria

All traceable patients prescribed sertindole after its market suspension were included. There were no exclusion criteria. Patients that started sertindole treatment before the market suspension (November 2^nd^, 1998) and continued to take it under the NPU programme constitute the "Before" group. Patients prescribed sertindole after the market suspension constitute the "After" group.

### Data collection

All physicians were asked to complete a case report form for each patient identified as having received sertindole. The form was comprised of questions on demographics, start and stop dates of sertindole treatment, doses of sertindole, reasons for switch to and stopping of sertindole, antipsychotic treatment prior to and after sertindole treatment, and relapse status. Information regarding occurrence of serious adverse events (SAEs) from the start date of sertindole and until 30 days after stopping sertindole, with cause and date, and cause and date of eventual death, was also included in the form. Copies of the case report form are available from the authors upon request.

Exposure was computed either from the date of start of sertindole treatment to the date of discontinuation (for the patients who stopped sertindole), or from the date of start of sertindole treatment to the date of last visit (for the patients still taking sertindole at the end of study).

The study period was from the date of first prescription of sertindole to 30 days after the end of the last prescription. All serious adverse events (SAEs) falling under International Committee on Harmonization (ICH) criteria and occurring during the study period were recorded and classified according to ICH definition.

SAEs were broken down into nine groups:

• Neuroleptic Malignant Syndrome

• QTc prolongation (calculated using Bazett's formula)

• Heart rate anomaly

• Syncope

• Convulsion

• Overdose

• Suicide attempt

• Death

• Other SAEs

Each case report was checked for data quality and clarified as needed. Overall, 461 data clarifications were issued. All but 45 (10%) were returned. These should however not alter the results, since they mainly concerned treatments before and after sertindole treatment.

Deaths were classified as suicide, sudden death, or other causes. Each classification was based on clinical review of death descriptions including all available documentation.

### Statistical analysis

The statistics were essentially descriptive: continuous variables were expressed as mean ± standard deviation (SD), while qualitative data were described using frequency and percentage. SAE occurrence rates were computed per number of patients (crude rate), and the total per person-years of exposure (PYE). The all-cause mortality rates were compared to those observed in the consolidated clinical trial database. The bilateral mortality rate 95% confidence intervals (CI) were calculated using Poisson's distribution.

Statistical analyses were performed using SAS software version 8.2 from SAS Institute, Cary, North Carolina USA.

Local regulatory rules were followed in each participating country. Where applicable, the study was submitted to and approved by the relevant authorities and/or the local Ethics Committee or Institutional Review Board.

## Results

1,444 case report forms were returned including 12 duplicates. These duplications primarily resulted from the same patient being followed – either consecutively or simultaneously – by more than one prescriber. Data on theses patients were consolidated after case revision. 1,432 patients were included in the study: Austria (8.9%), Belgium (10.9%), the Czech Republic (6.7%), Estonia (1.9%), Finland (6.0%), Germany (13.4%), Hungary (16.7%), Latvia (15.2%), The Netherlands (5.2%), Norway (7.5%) and Switzerland (7.8%). Average age (± SD) was 43.6 ± 15.0 for females and 37.3 ± 13.1 for males (44.8% of patients) (Table 1).

**Table 1:** Demographic Data

	Number of patients (%)*	Age (Years)**	
		
		**Mean ± SD**	**Quartiles (Q1 – Q3)**
Male	637 (44.8)	37.3 ± 13.1	27 – 45
Female	786 (55.2)	43.6 ± 15.0	33 – 53

Total	1,423 (100)	40.9 ± 14.5	30 – 49

* Gender was missing for 9 patients

**Among the 1,423 patients, age was missing for 1 patient

Just before the initial prescription of sertindole, more than half of the patients (666 of 1295, 51.4%) for whom prior antipsychotic information was available were treated with only typical antipsychotic(s), 32.4% (419 of 1295) with only atypical antipsychotic(s), and 10.6% (137 of 1295) with both typical and atypical antipsychotics. 5.6% of patients had no antipsychotic treatment prior to initial sertindole treatment. Most patients treated with an antipsychotic before sertindole were in monotherapy and receiving only one typical (54.3%) or one atypical (45.7%) antipsychotic (Table 2). Among patients treated with atypical antipsychotics only, patients receiving two or more atypical antipsychotics were few (4.5%), but it was common to be treated with two or more typical antipsychotics (54.9%) in patients treated with typical antipsychotics only. Before sertindole initiation, the most often prescribed antipsychotics were haloperidol (17.8%), risperidone (14.2%), clozapine (11.3%) and olanzapine (9.1%). These four drugs represented more than half of the antipsychotics taken prior to sertindole initiation.

**Table 2:** Number and Type of Antipsychotic Prescribed just Prior to Initial Prescription of Sertindole

**Number of antipsychotics**	**Total**	**Only typical antipsychotic(s)**	**Only atypical antipsychotic(s)**	**Both types**
	**N^a ^(%)**	**N^a ^(%)**	**N^a ^(%)**	**N^a ^(%)**
None	73 (5.6%)	-	-	-
1 antipsychotic	876 (67.6%)	476 (54.3%)	400 (45.7%)	-
2 antipsychotics	271 (20.9%)	151 (55.7%)	17 (6.3%)	103 (38.0%)
3 antipsychotics and more	75 (5.8%)	39 (52.0%)	2 (2.7%)	34 (45.3%)

Total	1,295 (100.0%)	666 (54.5%)^b^	419 (34.3%)^b^	137 (11.2%)^b^

^a ^For 137 patients, the name of the antipsychotic given before sertindole initiation was missing.

^b ^These percentages were calculated using a denominator of 1,222 (i.e., the total number of patients [1,295] minus those not initially taking an antipsychotic [73])

One thousand and thirty nine patients were recorded in the "Before" subgroup, and 393 in the "After" subgroup. The main reasons for prescribing sertindole and maintaining its use after its market suspension were lack of efficacy (49% in the "Before" group and 57.2% in the "After" group) or adverse events (25.5% in the "Before" group and 18.8% in the "After" group) from previous treatments (Table 3). The most frequent subgroups of adverse events reported under previous antipsychotic treatment were neurological disorders (51.4%) and metabolism and nutrition disorders (15.9%). The mean daily sertindole dosage was 13.4 ± 5.5 mg. 26.1% of patients received less than 12 mg, 73.3% received 12 mg to 24 mg, and 0.6% received more than 24 mg.

**Table 3:** Reasons for Prescribing Sertindole Treatment

	**Number of patients (%)**	
**Reasons**	"Before" subgroup (n = 1,039)*	"After" subgroup (n = 393)**

Lack of efficacy of previous treatment	498 (49.0%)	222 (57.2%)
Adverse event under previous treatment	259 (25.5%)	73 (18.8%)
Patient desire to continue	146 (14.4%)	41 (10.6%)
Non compliance to previous treatment	74 (7.2%)	27 (7.0%)
Other reasons	40 (3.9%)	25 (6.4%)

**Total**	**1,017 (100.0%)**	**388 (100.0%)**

* This group consisted of patients who started taking sertindole before the market suspension and who continued to take it during the suspension; information was missing for 22 patients

** This group consisted of patients who started taking sertindole during the market suspension; information was missing for 5 patients

At the time of their last physician visit, more than half of the patients were still being treated with sertindole. The average treatment duration for patients who were still treated with sertindole at the date of the last visit was 19.2 ± 8.3 months. The average treatment duration for patients who stopped treatment with sertindole was 11.9 ± 7.2 months. 682 patients in total from the "Before" and "After" groups stopped sertindole treatment. The primary reason for this was related to its market suspension and the related administrative burden to get access to the NPU programme; this accounted for about one third of the cases of patients who stopped sertindole. Lack of efficacy of sertindole was the reason for discontinuing for 12.8% of patients in the "Before" subgroup and 21.3% in the "After" subgroup (Table 4). Among patients who discontinued sertindole treatment, 64.0% of patients had stopped for more than 6 months at the date the form was completed. Almost one third of these patients had relapsed within 6 months following sertindole discontinuation. After stopping sertindole treatment, 62.0% of patients were prescribed treatment with at least one atypical antipsychotic with or without typical antipsychotics, and 22.3% of patients were switched to only typical antipsychotics.

**Table 4:** Reason for Sertindole Discontinuation during Study Follow-up (682 patients), with type of previously taken treatment

	Total N (%)	
**Reason for Stopping**	**Before**^a^	**After**^b^

**Sertindole suspension and Named Patient Use restrictions**	**176 (35.3%)**	**49 (26.8%)**
Typical antipsychotic(s)^c^	50 (25.1%)	28 (31.5%)
At least one atypical antipsychotic^d^	49 (31.6%)	15 (20.8%)

**Patient desire to stop**	**127 (25.5%)**	**50 (27.3%)**
Typical antipsychotic(s)^c^	71 (35.7%)	26 (29.2%)
At least one atypical antipsychotic^d^	39 (25.2%)	18 (25.0%)

**Lack of efficacy**	**64 (12.8%)**	**39 (21.3%)**
Typical antipsychotic(s)^c^	28 (14.1%)	17 (19.1%)
At least one atypical antipsychotic^d^	23 (14.8%)	19 (26.4%)

**Adverse Event(s)**	**58 (11.6%)**	**19 (10.4%)**
Typical antipsychotic(s)^c^	26 (13.1%)	10 (11.2%)
At least one atypical antipsychotic^d^	20 (12.9%)	9 (12.5%)

**Other reasons**	**74 (14.8%)**	**26 (14.2%)**
Typical antipsychotic(s)^c^	24 (12.1%)	8 (9.0%) 11
At least one atypical antipsychotic^d^	24 (15.5%)	(15.3%)

**Total**	**499 (100.0%)**	**183 (100.0%)**

^a ^For 2 patients, reason for stopping sertindole treatment was missing.

^b ^For 1 patient, reason for stopping sertindole treatment was missing.

^c ^For 129 patients, the name of the antipsychotic given before sertindole treatment was missing and 48 patients had no antipsychotic treatment before sertindole.

^d ^For 8 patients, the name of the antipsychotic given before sertindole treatment was missing and 25 patients had no antipsychotic treatment before sertindole.

A total exposure of 1,759 PYE was calculated for patients who stopped or continued sertindole treatment. Exposure was also computed by taking into account exposure occurring after sertindole suspension (i.e. observation period of the survey). The mean per patient exposure was 1.23 PYE.

### Safety and serious adverse events

10 of the 97 SAEs recorded had a fatal outcome. There were 2 suicides, 3 sudden deaths (2 myocardial infarctions, 1 pulmonary embolism), 4 "other" deaths (1 pulmonary embolism; 1 unspecified intoxication; 1 myocardial infarction; 1 mesenteric artery infarction), and 1 unascertained cause of death.

The all-cause mortality rate was 0.51 per 100 PYE, with a 95% Poisson confidence interval of [0.23–0.97]. When considering only exposure after market suspension of sertindole (1,396 PYE), the death rate was 0.64 per 100 PYE, with a 95% Poisson confidence interval of [0.30–1.22]. The death rates per aetiology are described in Table 5.

**Table 5:** Mortality rates per aetiology*

	***Suicide***	***Sudden death***	***Other causes***	***Total deaths***
**Number of deaths**	**2**	**3**	**4**	**9**

**Total Exposure**				

Death Rate per 100 PYE	0.11	0.17	0.23	0.51
95% Poisson CI	[0.01–0.41]	[0.04–0.50]	[0.06–0.58]	[0.23–0.97]

**Exposure after Market Suspension**				

Death Rate per 100 PYE	0.14	0.21	0.29	0.64
95% Poisson CI	[0.02–0.52]	[0.04–0.63]	[0.08–0.73]	[0.30–1.22]

* 1 death was unascertained and is therefore not included in this table

PYE: Patient Year Exposure

The remaining 87 SAEs were non-fatal (Table 6). Among these, 24 were cardiac SAEs (QT prolongation was reported in 15 patients, there were 1 abnormal ECG, 4 heart rate anomalies, 3 cardiac failures, and 1 myocardial infarction). Despite observed QT prolongation with sertindole, there were no recorded cases of *torsades de pointes *in this survey. QT prolongation was reported in 10 patients in the "Before" subgroup, accounting for 0.70% of total patients (Rate/100 PYE 0.67 [95%CI: 0.32–1.23]). In the "After" subgroup, QT prolongation was reported in 3 patients, accounting for 0.21% of total patients (Rate/100 PYE 1.15 [95%CI: 0.24–3.37]). Detailed information on QT prolongation was missing for 2 patients (Table 7).

**Table 6:** Most Frequent Non-fatal Serious Adverse Events

	**Number**	**% of total patients**	**Rate/100 PYE (95% CI)**
QT_C _prolongation	15	1.05	0.85 (0.48–1.41)
Suicide attempts/Other overdoses	7	0.49	0.40 (0.16–0.82)
Heart Rate Anomaly	4	0.28	0.23 (0.06–0.58)
Convulsion	4	0.28	0.23 (0.06–0.58)
Overdose of sertindole	4	0.28	0.23 (0.06–0.58)
Syncope	3	0.21	0.17 (0.04–0.50)
Carcinoma/Tumour	2	0.14	0.11 (0.01–0.41)
Other serious adverse event	48	3.35	2.73 (2.01–3.62)

**Total**	**87**	**6.08**	**4.95 (3.96–6.10)**

**Table 7:** Most Frequent Non-fatal Serious Adverse Events – QTc prolongation

	**Number**	**% of total patients**	**Rate/100 PYE (95% CI)**
**QT_C _prolongation**	15 *	**1.05**	**0.85 (0.48–1.41)**
"Before" group	10	0.70	0.67 (0.32–1.23)
"After" group	3	0.21	1.15 (0.24–3.37)

* Among the 15 cases, only 14 CIOMS reports (Council for International Organizations of Medical Sciences) were retrieved; the initiation date of one case was not available.

## Discussion

This was a retrospective multicentre survey of all known patients treated by sertindole in 11 European countries. The objective was to gather information on all patients treated with sertindole in Europe after its market suspension, and to assess the modality of sertindole prescription in this population and to assess serious adverse events that could be related to the use of sertindole. While the restrictions imposed by the named patient use programme allowed for a high level of exhaustivity in the study, they also meant that prospective studies involving randomisation and blinding were impossible. Our discussion and conclusions are thus based on comparisons with available literature alone.

Exhaustiveness was the goal in the identification of patients treated with sertindole. In some countries, this was possible since all patients who had ever received sertindole received it through a specific named-patient use programme. Exhaustive data was obtained from 72% of patients treated in Austria to more than 90% in Belgium, Estonia, Hungary, and the Netherlands. In Germany and Hungary, patients treated with sertindole were followed prospectively and exhaustively through specific protocols. In Latvia and Norway, exhaustiveness could not be assessed. The study was thus limited by the fact that not all records of patients treated with sertindole were studied. In addition, the market suspension of sertindole and its prescription on the NPU basis would have had an effect on the profile of the patients being treated, with those patients more at risk of cardiac events being discontinued. This may have had an effect on results. However, it is unlikely that SAEs were missed in this survey, due to the close follow up with physicians.

The two primary reasons for prescribing sertindole treatment after its market suspension were lack of efficacy of previous antipsychotic treatment for half of the patients, and occurrence of adverse events under previous antipsychotic treatment for about 20% of the patients. More than half of the patients had received a typical antipsychotic and one third had received atypical antipsychotics before sertindole treatment. More than one quarter of these patients received 2 or more antipsychotics at the same time before initiation with sertindole, which is in line with rates reported in the literature [18,19].

The mean exposure to sertindole per patient in this study was high, greater than one year (1.23 PYE). This result should be considered in the light of the CATIE study results [12], where the longest time to discontinuation of an atypical antipsychotic treatment for any causes was 9.2 ± 3.1 months, whereas the treatment duration for patients treated with sertindole who stopped due to any cause was 11.9 ± 7.2 months. In fact, the observed average exposure in our study underestimates real exposure, since approximately 52% of the patients were still being treated with sertindole at the date of their last visit to the physician. The low discontinuation rates due to lack of efficacy (15.1%) or intolerability (11.3%) may be possible explanations for the long mean exposure, which was the longest to be reported for sertindole treatment.

Patient desire to stop accounted for 26.0% of discontinuations. It is not clear exactly why these patients chose to stop treatment, but this could have been due to adverse publicity surrounding the drug, the market suspension itself, or due to unvoiced adverse events.

Among patients who discontinued sertindole (with a follow-up greater than 6 months), 31.8% experienced relapse. According to a literature review, the expected one-year relapse rate ranges from 16.2% [20], to 35.4% [21]. Thus, the observed relapse rate following sertindole discontinuation appeared to be in the higher range previously reported.

All-cause mortality in the present study (0.51 per 100 PYE [95% CI: 0.23–0.97]) was lower than that observed in other post-marketing studies [22]. This probably reflects the fact that patients were carefully selected and followed by physicians. However, the patients who died had mostly received multiple therapy prior to start of the study, which has been indicated in the literature to be associated with reduced survival [23].

The rate of non-fatal SAEs was 4.95 per 100 PYE. The primary SAEs were psychosis (related to the course of the disease) and QT prolongation (15 cases, 0.85 per 100 PYE). QT prolongation included 6 cases where the QT interval increased but there were no clinical effects. Since regular ECG monitoring was performed, it was expected that a higher QT prolongation rate would be found.

## Conclusion

The temporary marketing suspension of sertindole involved considerable medical and administrative restrictions on its supply and use, and made normal study procedures impossible to follow. However, comparing our results with available literature, sertindole appeared to have an equivalent or favourable profile to other antipsychotic drugs when taking into account mean exposure, relapse rate, and the all cause mortality rate. The high mean exposure supports the appropriateness of sertindole in specific cases. The lifting of the market suspension means that sertindole is now an option in the disease management of schizophrenia.

## Competing interests

CL has received grants from and acted as a consultant to: Bristol Meyers Squibb, Eli Lilly & Co., H. Lundbeck A/S, Janssen Pharmaceuticals and Sanofi-Aventis. MT was formerly employed by H. Lundbeck A/S, the manufacturer of sertindole. CS and KH are employees of H. Lundbeck A/S, the manufacturer of sertindole.

## Authors' contributions

CL participated in the study design and helped to draft the manuscript. CS collected the data, performed the statistical analysis, and drafted the manuscript. MT and KH participated in the study design and statistical analysis. All authors read and approved the final manuscript.

## Pre-publication history

The pre-publication history for this paper can be accessed here:


